# Hypersaline Lake Urmia: a potential hotspot for microbial genomic variation

**DOI:** 10.1038/s41598-023-27429-2

**Published:** 2023-01-07

**Authors:** Roohollah Kheiri, Maliheh Mehrshad, Ahmad Ali Pourbabaee, Antonio Ventosa, Mohammad Ali Amoozegar

**Affiliations:** 1 Extremophiles Laboratory, Department of Microbiology, School of Biology and Center of Excellence in Phylogeny of Living Organisms, College of Science, University of Tehran, Tehran, Iran; 2 Department of Aquatic Sciences and Assessment, Swedish University of Agricultural Sciences, 750 07 Uppsala, Sweden; 3 Department of Soil Science, Agriculture Engineering and Technology, College of Agriculture and Natural Resources, University of Tehran, Karaj, Iran; 4 Department of Microbiology and Parasitology, Faculty of Pharmacy, University of Sevilla, 41012 Sevilla, Spain

**Keywords:** Metagenomics, Metagenomics, Metagenomics

## Abstract

Lake Urmia located in Iran is a hypersaline environment with a salinity of about 27% (w/v). Metagenomic analyses of water samples collected from six locations in the lake exhibited a microbial community dominated by representatives of the family *Haloferacaceae* (69.8%), mainly those affiliated to only two genera, *Haloquadratum* (59.3%) and *Halonotius* (9.1%). Similar to other hypersaline lakes, the bacterial community was dominated by *Salinibacter ruber* (23.3%). Genomic variation analysis by inspecting single nucleotide variations (SNVs) and insertions/deletions (INDELs) exhibited a high level of SNVs and insertions, most likely through transformation for abundant taxa in the Lake Urmia community. We suggest that the extreme conditions of Lake Urmia and specifically its high ionic concentrations could potentially increase the SNVs and insertions, which can consequently hamper the assembly and genome reconstruction from metagenomic reads of Lake Urmia.

## Introduction

Hypersaline environments, widely distributed across a variety of climate zones, are characterized by high salt concentration and are often aquatic systems (thalassohaline, of marine origin or athalassohaline, formed by dissolution of mineral salt deposits of continental origin) or saline soils^1, 2^. Studies on different hypersaline environments show that *Haloquadratum* and certain *Balneolaeota* members may preferably grow in aquatic or soil habitats, respectively, while haloarchaea, nanohaloarchaea, and *Salinibacter* are capable of adapting to both environments^3^. Microbial cells require specific adaptations that enable them to thrive in the extreme conditions of different hypersaline environments^4^. In addition to their intrinsic capabilities, microorganisms may undergo variations to adapt to extreme conditions. Generally, mutation and lateral gene transfer in microbial communities are instrumental in developing adaptive features or fitness-conferring variants^5^. The balance of variation and selection causes the unadapted cells to be outcompeted by thriving microorganisms that are getting selected, which could consequently lead to reduced phylogenetic diversity of the community and potentially an increased rate of variation at the strain level^4^.

Metagenomic studies, focused on the diversity and metabolic capabilities of reconstructed metagenome-assembled genome (MAGs) from environmental samples, have expanded our knowledge about microbial diversity of natural ecosystems, including hypersaline environments^6, 7^. However, these studies overlook the within-species diversity of microbial inhabitants of these environments. In ecosystems with high microbial diversity at strain level (microdiversity), where the performance of MAG reconstruction is limited, we need to adopt elaborate approaches to study within-species gene content variations, single nucleotide variations (SNVs), and INDEL profiles^8^.

Our knowledge of prokaryotic microdiversity is largely based on comparative genomic analyses of isolates from different locations. Originating from completely different habitats, it is expected for these genomes to vary in fitness backgrounds and genomic adaptations specific to the local conditions. To evaluate the gene content of strains and variations in these genes, pan-genome studies can present valuable information. Pan-genome contains both the core that is present in all organisms belonging to the same species as well as accessory genes, which are not shared in all representatives but could potentially confer important additional capabilities^9^.

In this study, we explore the microdiversity and pan-genome of highly abundant microorganisms in the hypersaline Lake Urmia, an endorheic salt lake located in Northwest Iran. At its greatest extent, Lake Urmia was the largest lake in the Middle East and the sixth-largest hypersaline lake on Earth^10^. Despite its distance from the sea, based on palaeogeographic studies, the lake has marine origin as a remnant of the Paratethys sea, which started to dry from the Pleistocene epoch, leaving Lake Urmia, Aral and the Caspian Sea^11^. In terms of salinity, Lake Urmia is characterized by an extreme salinity, about 27% (w/v), with a high level of Cl^−^, Na^+^, SO_4_^2−^, Mg^2+^, K^+^, and Ca^2+^ ions. Several studies have been performed to explore the microbial diversity of Lake Urmia by using cultivation and 16S rRNA cloning and sequencing approaches^12–14^. However, these studies are limited in providing a fine-scale and comprehensive insight into its microbial diversity. Therefore, we present an overview of the metagenome-assembled genomes and genetic variation of the microbial community of this lake. Using metagenomic data, we evaluated the diversity and composition of its prokaryotic community and studied the SNVs of the abundant microbial taxa in Lake Urmia.

## Results and discussion

### Physico-chemical features of Lake Urmia

**Figure 1:**
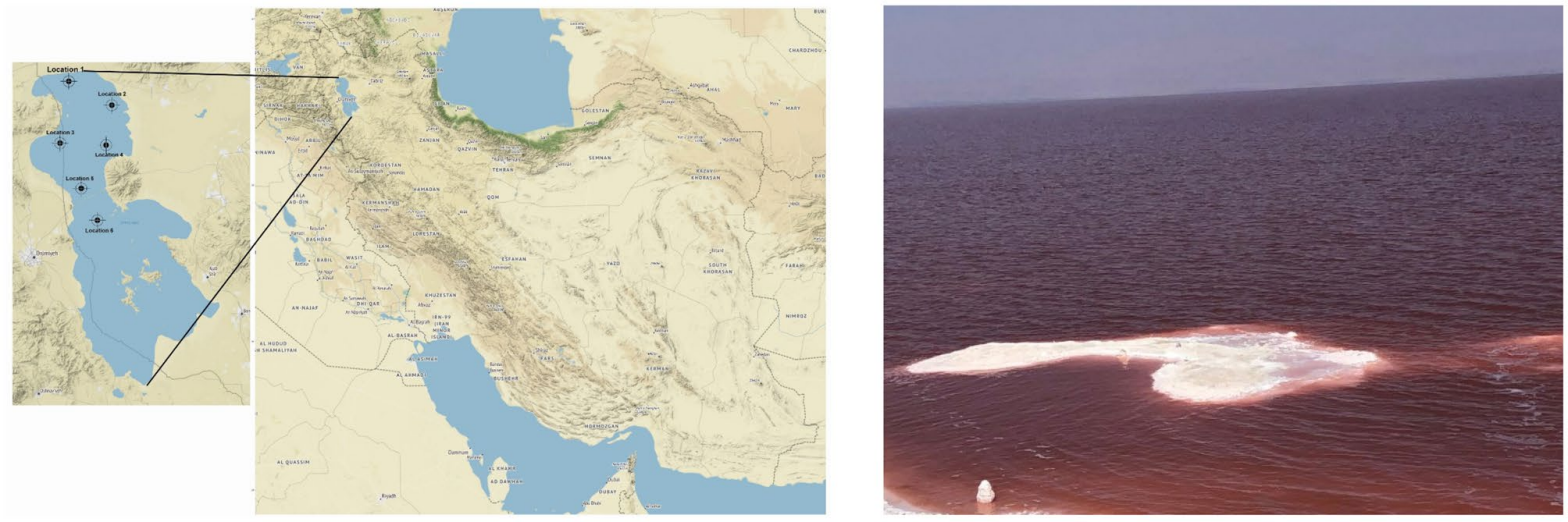
Location of Lake Urmia in Iran and of the six sampling sites. The map was prepared in R studio, using the ggmap 3.0.1 package (left). View of hypersaline Lake Urmia on October 20th, 2020 (right).

Sampling was performed during the period of lowest rainfall and input volume in the year when the lake water reached the highest salt concentration (locations shown in Fig. 1, Supplementary Table S1). The measured ionic composition of the collected brine showed the typical composition of halite-dominated thalassohaline brines, rather than the continental ones. Concentrations of major anions and cations are shown in Table 1. Six major ions including Cl^−^ (180,000 mg/l), Na^+^ (92,500 mg/l), SO_4_^2−^ (25,260 mg/l), Mg^2+^ (17,750 mg/l), K^+^ (4000 mg/l), and Ca^2+^ (512 mg/l) comprised more than 99.9% of the ionic composition. This ionic composition suggests evaporation, calcium sulfate, and calcium carbonate precipitation as the main causes of the salt concentration, and high electrical conductivity (EC), total dissolved solids (TDS), hardness, and alkalinity of the lake water. The pH of the lake was 8.9, resulting in an alkaline and more extreme habitat. In terms of the microelements, only zinc (Zn) was detected, while iron, lead, copper, and cadmium concentrations were below the detection limit of our method (Table 1).

**Table 1:** Physico-chemical characteristics of the Lake Urmia.

Parameter	Unit	Measuring method	Location 1	Location 2	Location 3	Location 4	Location 5	Location 6	Average
EC	μmhos/cm	SM 2510-B	513,200	512,800	511,600	509,200	511,200	504,000	510,333.33
pH		SM 4500-H-B	8.9	8.97	8.96	8.84	8.93	8.94	8.92
Turbidity	NTU	SM 2130-B	23.2	20.6	33.8	28.4	20.4	24.8	25.2
TDS	mg/l	SM 2510	333,620	333,280	332,480	331,000	332,260	327,560	331,700
Total alkalinity	mg/l CaCO_3_	SM 2320-B	1368	1360	1336	1360	1360	1248	1338.67
Total hardness	mg/l CaCO_3_	SM 2340-C	90,000	84,000	90,000	90,000	90,000	71,200	85,866.67
Sodium (Na^+^)	mg/l	DIN: 6919	97,600	93,800	93,000	90,000	90,400	90,200	92,500
Calcium (Ca^2+^)	mg/l	DIN: 6919	512	518	510	520	510	506	512.67
Magnesium (Mg^2+^)	mg/l	DIN: 6919	17,850	17,850	17,650	17,600	17,700	17,750	17,733.33
Potassium (K^+^)	mg/l	SM 3500-K	4400	4200	4000	4000	4000	3400	4000.00
Chloride (Cl^−^)	mg/l	SM 4110-B	180,340	180,340	180,340	180,340	180,340	178,920	180,103.33
Sulfate (SO_4_^2−^)	mg/l	SM 4110-B	25,500	25,000	25,500	25,600	25,500	24,500	25,266.67
Nitrate (NO_3_^−^)	mg/l	SM 4110-B	14.4	15	14.4	14.4	14.6	12	14.13
Fluoride (F)	mg/l	SM 4110-B	17	18.4	17.6	18.6	18.4	12.4	17.07
Iron (Fe)	mg/l	SM 3500Fe-B	0.78	1.76	1.04	0.78	1.16	1.18	1.12
Lead (Pb)	μg/l	DIN:38406	< 1	< 1	< 1	< 1	< 1	< 1	–
Copper (Cu)	μg/l	DIN:38406	< 1	< 1	< 1	< 1	< 1	< 1	–
Cadmium (Cd)	μg/l	DIN:38406	< 0.1	< 0.1	< 0.1	< 0.1	< 0.1	< 0.1	–
Zinc (Zn)	μg/l	DIN:38406	11.56	12.42	13.17	12.91	14	14.1	13.03
TOC (total organic carbon)	μg/l	SM 5310B	< 60	< 60	< 60	< 60	< 60	< 60	< 60

*SM*, Standard Methods for the Examination of Water and Wastewater, 23rd Edition.

*DIN*, German Institute for Standardization.

### Overall microbial diversity of Lake Urmia

**Figure 2:**
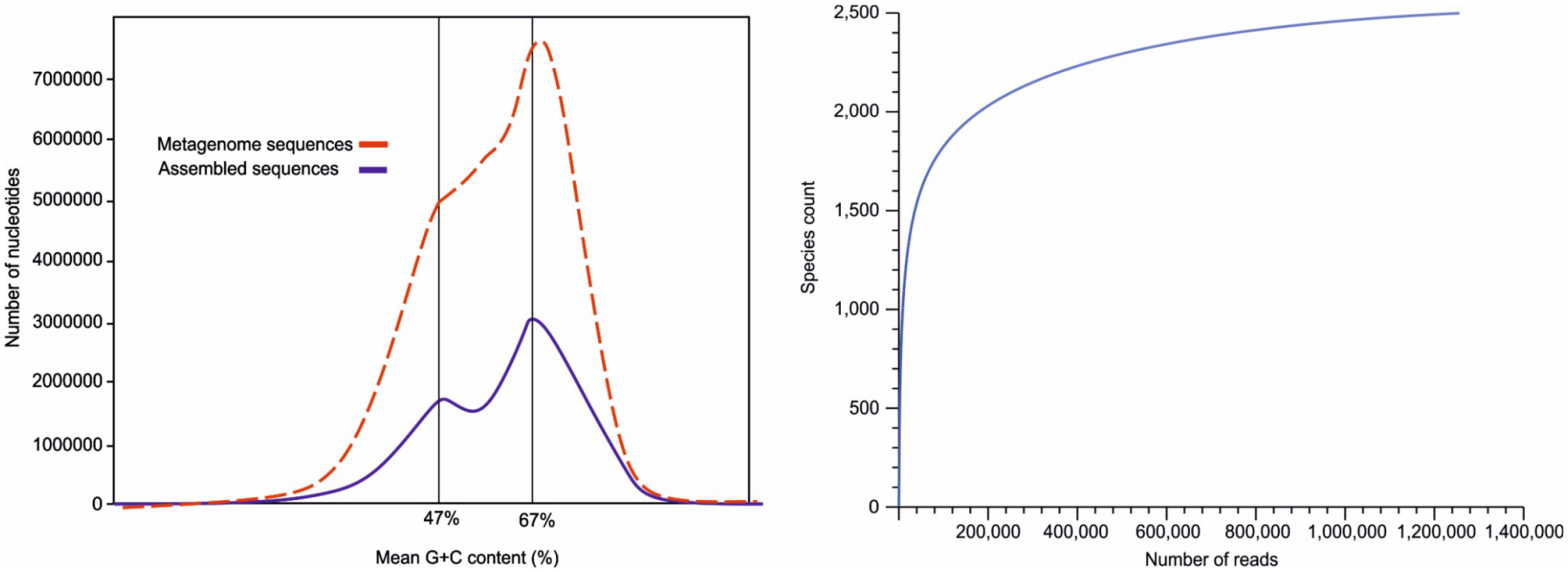
Percentage of the G+C content of the raw reads and assembled metagenome sequences (left). Rarefaction plot of the phylogenetic diversity of the metagenomic reads (right).

The distribution of peaks in a length-weighted G+C histogram showed that the G+C content of the raw metagenomic reads and assembled contigs in reconstructed LUMs are consistent. As shown in Fig. 2, discrete peaks at 47% and 67% G+C content of raw metagenomic reads correspond to the G+C content of the reconstructed LUMs (Supplementary Table S2), including *Haloquadratum* at 47% G+C, and several archaeal and bacterial populations with G+C content at around 67%.

Taxonomic profiling of the merged paired-end sequences (~ 204 million raw reads) using MetaPhlAn3^15^ showed that Archaea dominate the community, matching 76.53% of the reads (composed of 69.88% *Euryarchaeota* and 6.65% unclassified phyla). A total of 23.46% of reads match Bacterial reference genomes (composed of 23.33% *Bacteroidota* and 0.13% *Pseudomonadota*) (Supplementary Table S3).

Barrnap v0.9^16^ retrieved a total of 157 16S rRNA gene sequences (398–1559 bp in length) from the assembly: among which 70, 52, and 12 sequences were affiliated to the Archaeal orders *Halobacteriales, Haloferacales*, and “*Nanosalinales*”, respectively, while the Bacterial 16S rRNA sequences were mostly affiliated with the family *Rhodothermaceae* (Supplementary Table S4; Supplementary Fig. S1).

### Genome-resolved analysis of the Lake Urmia microbial diversity

The presence of closely related strains of the same species in the ecosystem causes high genetic variation leading to insufficient coverage of each strain that might hamper assembly and consequently MAG reconstruction for such groups^17^. In this study, 204 million sequenced high-quality (Phred quality score > 30) paired-end reads (150 bp), provided acceptable depth but the different abundance of various species in a metagenomic sample resulted in a highly non-uniform read coverage across different genomes. We detected an outstanding level of variations (arising from SNVs and insertions) in the dominant microorganisms, including representatives of the haloarchaeal genera *Haloquadratum* and *Halonotius*, and the extremely halophilic bacterium *Salinibacter ruber*, which is causing a decrease in the completeness of their MAGs reconstructed from the metagenome of Lake Urmia. Binning assembled contigs (Full Quast report of the assembly is provided in Supplementary Table S5) longer than 2.5 kb produced 80 LUMs, among which 22 LUMs (CheckM estimated completeness ranging from 44.88 to 97.15%) were used for further analysis.

Largely consistent with the results from Barrnap v0.9, mapping metagenomic reads to the reference genomes showed that the most prevalent species of the Lake Urmia was *Haloquadratum walsbyi* DSM 16790, followed by *Halonotius pteroides* CECT 7525, and the most abundant LUMs were LUM.12 and LUM.22, affiliated with the family *Haloferacaceae* and *Haloquadratum walsbyi,* respectively. Further results of the coverM (0.6.1) are illustrated in Supplementary Fig. S2 and Supplementary Table S6. Our results showed that 71.77% of the raw metagenomic reads were mapped to sequences in the reference genome, while 28.23% of the sequenced reads remained unmapped. The distribution of different LUMs and reference genomes are shown in Supplementary Fig. S2.

### Genetic variation of archaeal and bacterial LUMs

**Figure 3:**
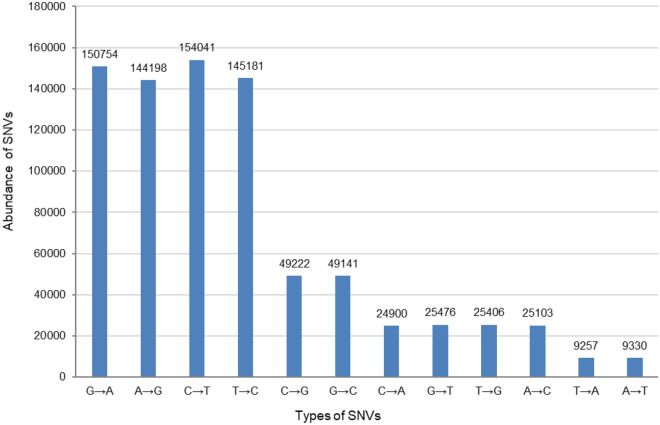
Frequency of all detected SNV types in LUMs and reference genomes with > 1% relative abundance in the metagenome.

We identified 812,009 SNVs of all possible point variations (transition and transversion) in the LUMs and reference genomes with > 1% relative abundance in the metagenome. These variations were prevalent, especially in *Haloquadratum*, *Salinibacter*, and *Halonotius* representatives (the number of SNVs is shown in Fig. 3 and a full description is provided in Supplementary Table S7). In the case of INDELs, the frequency of deletions was low, while insertions were prominent. The length of the insertions ranged up to 354 nucleotides and was frequent in all reads mapped to the reference genomes, especially in *Haloquadratum, Salinibacter*, and *Halonotius* representatives (Supplementary Table S8).

DNA sequence variation can be introduced from internal sources (within the cell) during metabolic processes, generating oxidation, hydrolysis, and alkylation damage, along with the incorporation of mismatched bases^18^. Further, inducing agents such as UV and ionizing radiation as well as various chemical mutators may cause the incorporation of base analogs and base lesions, including the deamination, depurination, and methylation of bases, oxidative damage, and DNA double-strand breaks (DSB)^19^. If the repairing mechanisms work well, these are excluded, but if unsuccessful, an SNV is perpetuated; for example, among DSB repair pathways, there are non-homologous end joining and microhomology-mediated end joining, which respond quickly with a high error rate, causing INDEL and translocations^18^. We hypothesize that high ionic levels of the hypersaline environment may contribute to the high SNVs. For prokaryotes with the “salt in” strategy, high levels of K^+^, Na^+^, and Mg^2+^ (and other ions) may interfere with the accuracy of archaeal enzymes and the associated chaperones leading to SNVs, however, further empirical proofs are required to confirm this hypothesis.

While the compaction and stabilization of DNA in eukaryotic cells are provided by the function of histone proteins (neutralizing 57% of the DNA negative charge)^20^, the remaining negative charge of DNA is compensated for by cations. The stability of dsDNA increases at higher salinities^21^. As reported by Borin et al.^22^, naked DNA in deep-sea anoxic hypersaline brines, independent of the species of origin, was capable of participating in natural transformation after weeks of exposure. In addition to their role in stabilizing the structure of DNA molecules, cations can increase the melting temperature of DNA molecules. GC-rich regions of the DNA also have a higher melting point. Altogether, it is quite expected that intact, active, and double-stranded standard DNA (which has higher stability compared to single-stranded DNA) from disrupted cells is present in Lake Urmia. A combination of cations as mediators, together with the lower temperature of the lake (the average annual temperature is 9 °C) facilitates the suitable folding of DNA into a compact structure. Additionally, it mediates the attachment of the DNA to the negative charges of the cell membrane’s phosphatidylcholine and phosphatidylserine, DNA-uptake apparatus, anchoring and stabilizing the interaction of DNA with the membrane^23^. Furthermore, the calcium ions bound to the cell membrane also cause changes in the membrane permeability and facilitates DNA entry into the cell^22^. In addition to standard B-DNA, research has shown that the presence of GC-rich DNA molecules in saline solutions can cause non-standard structures of G-quadruplexes and G-triplex, which have much higher resistance than the standard DNA; however, their role in transformation is not well studied^24^.

As the most dominant prokaryotes of the lake and based on the extent of coverage, breadth, SNV, and INDEL, we focused on the dominant taxa in the community using reference genomes including *Haloquadratum walsbyi* DSM 16790*, Salinibacter ruber* ST67, their corresponding LUMs, and *Halonotius pteroides* CECT 7525.

### Genus *Haloquadratum*

**Figure 4:**
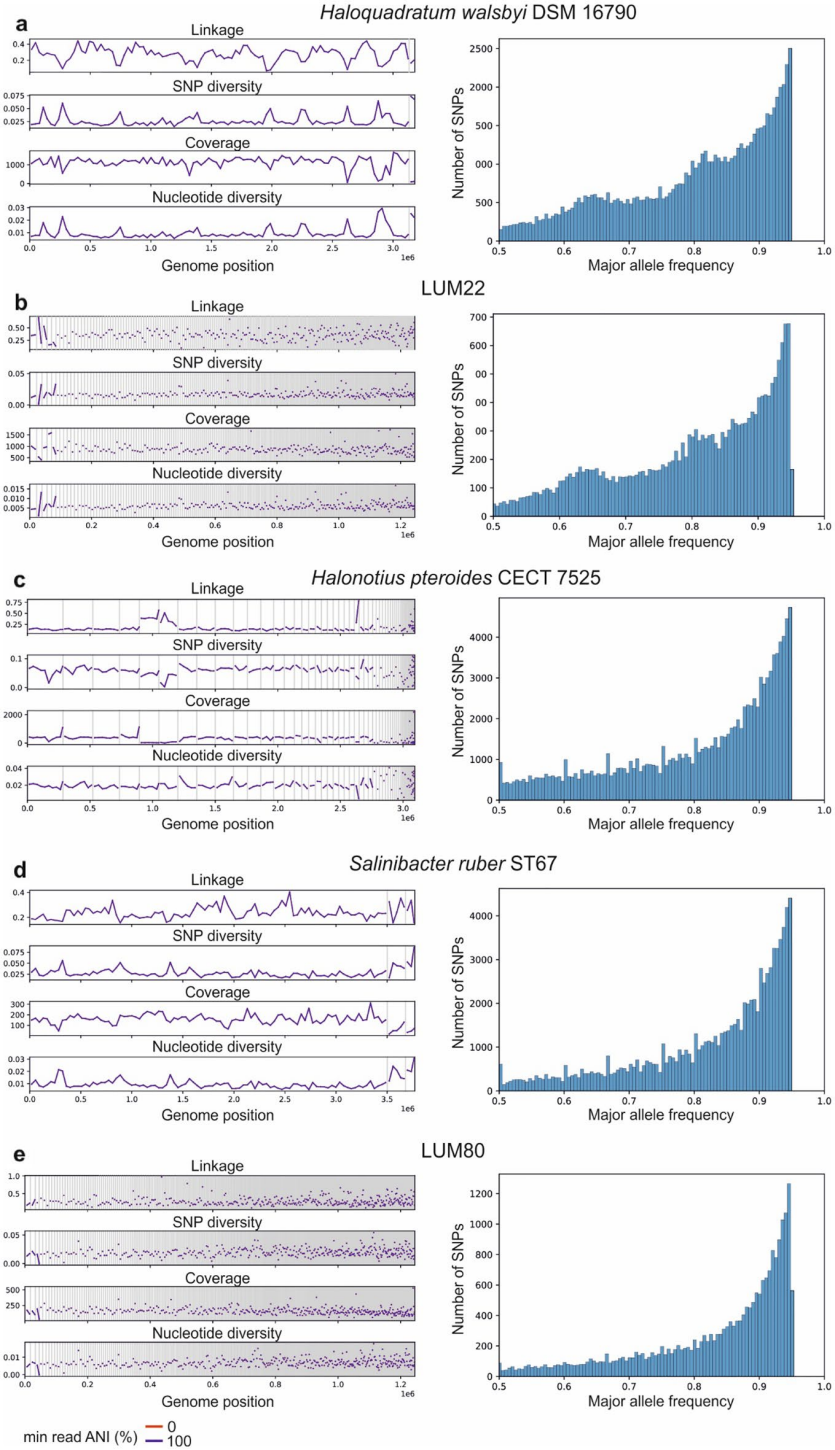
Genetic variation (SNP diversity, coverage, and nucleotide diversity) of *Haloquadratum walsbyi* DSM 16790 (**a**; left), LUM.22 (**b**; left), *Halonotius pteroides* CECT 7525 (**c**; left), *Salinibacter ruber* ST67 (**d**; left), LUM.80 (**e**; left). Distribution of major allele frequency of bi-allelic SNVs in *Haloquadratum walsbyi* DSM 16790 (~ 2500 SNVs with 95% major allele frequency) (**a**; right), LUM.22 (~ 700 SNVs with 95% major allele frequency) (**b**; right), *Halonotius pteroides* CECT 7525 (4000 SNVs with 95% major allele frequency) (**c**; right), *Salinibacter ruber* ST67 (~ 4000 SNVs with 95% major allele frequency) (**d**; right), LUM.80 (~ 1200 SNVs with 95% major allele frequency) (**e**; right).

A major percentage of the Lake Urmia metagenomic reads was mapped to *Haloquadratum walsbyi* DSM 16790 (14.53%), with a minor contribution of *Haloquadratum walsbyi* J07HQW2 (0.14%)*, Haloquadratum walsbyi* C23 (0.08%) and *Haloquadratum *sp. J07HQX50 (0.05%)*.* The square-shaped halophilic *Haloquadratum walsbyi*, with a G+C content of 47.9%, is a member of the family *Halobacteriaceae* and is the most prevalent archaeon found in saline environments as documented in previous studies^25^. This archaeon can grow optimally at 23–30% (w/v) total salt concentrations, is tolerant to very high magnesium levels, and as a photoheterotroph, it can obtain energy from light absorbed by bacteriorhodopsin^26^. Possessing two 3.1 Mb chromosomes, many deletions, short direct repeats (4–20 bp), and insertions have been reported by Dyall-Smith et al*.*^18^, conferring that the uptake and integration of foreign DNA have contributed to the evolution of *Haloquadratum walsbyi* genome. Consistent with GATK 4.2, which yielded a high density of genomic variation, a strain-level study of *Haloquadratum walsbyi* using inStrain 1.5.7, exhibits a high level of SNVs. Due to the high abundance of this archaeon, the high number of SNVs and reception of external DNA from not only intra-species but also inter-species sources, such as *Haloarcula* sp. CBA1115, *Haloplanus rubicundus*, or the halophilic archaeon DL31, the genetic diversity of this microorganism in Lake Urmia is very high. Based on peaks in the SNV distribution at about 63%, and 83%, it seems that there are two other strains present in the sample which can be justified due to the existence of LUM.47 and LUM.48 (Fig. 4a). Our results showed that most of the detected insertion sequences originated from *Haloquadratum walsbyi* C23. Moreover, a plasmid from *Haloplanus rubicundus*, a 63 bp insertion from the chromosome of the halophilic archaeon DL31, and two identical insertions of 223 bp from *Haloarcula* sp. CBA1115, and a 49 bp insertion from the chromosome of *Salinarchaeum* sp. IM2453 were also spotted in the metagenomic reads that mapped to the *Haloquadratum walsbyi* genomes.

LUM.22, LUM.47, and LUM.48 retrieved 11.69%, 0.25%, and 0.36% of the mapped reads, and are affiliated to *Haloquadratum walsbyi, Haloquadratum* sp., and *Haloquadratum walsbyi_*A, respectively. The completeness of these LUMs is respectively, 59.11%, 85.61%, and 73.77%. Numerous detected SNVs and INDELS have yielded a pool of genetic variation for *Haloquadratum walsbyi* in Lake Urmia leading to disruption of the assembly process and consequently low completeness of representative MAGs of highly abundant taxa in the lake.

The overall variation of LUM.22 is lower than that of *Haloquadratum walsbyi* DSM 16790 (Fig. 4b) since during the assembly, reads with SNV or insertion may not overlap to produce a contig and therefore will not be associated in the final LUM. Our results showed some insertions from *Haloquadratum walsbyi* and a 36 bp insertion from *Halorubrum* sp. PV6 plasmid in LUM.22.

### Genus *Halonotius*

Representatives of the next most abundant genus, include *Halonotius pteroides* CECT 7525 (6.8%), *Halonotius* sp. J07HN4 (1.2%), and *Halonotius* sp. J07HN6 (3.96%). *Halonotius* has a worldwide environmental distribution and has been reported in several habitats, such as solar salterns in Turkey^27^ and Spain^28^, saline lakes in Australia^29^, and China^30^, and food-grade salt samples^31^.

*Halonotius pteroides* CECT 7525 is among the reference genomes with the highest SNV frequency and insertion sequences. Our results showed a diverse microbial source for insertions in the *Halonotius pteroides* CECT 7525 genome, including plasmids (from *Natrinema* sp. YPL30, *Natrinema* sp. DC36, and *Haloprofundus* sp. SEDH52), chromosomes from *Salinibacter ruber*, *Haloquadratum walsbyi*, *Haloplanus rallus*, *Halapricum desulfuricans*, *Halalkaliarchaeum desulfuricum*, *Haloferax volcanii* DS2, *Halorubrum* sp., *Natronorubrum bangense*, and several other taxa. Consistent with these results, inStrain 1.5.7, provided significant nucleic diversity and SNVs signifying great variation in the *Halonotius* population (Fig. 4c). Despite their high abundance, no LUM with completeness ≥ 40% was reconstructed from representatives of this genus.

### Genus *Salinibacter*

*Salinibacter ruber* ST67 (1.19%), *Salinibacter ruber* M1 (0.88%), *Salinibacter ruber* SP2521 (0.68%), and *Salinibacter ruber* SP273 (0.67%), are reference representatives of the next most prevalent genus determined on metagenome of the Lake Urmia. Because of its broad distribution and high intraspecific genomic and functional diversity at both transcriptomic and metabolomic levels, this bacterium can be considered one of the main models for ecological and evolutionary studies of bacterial adaptation to hypersaline environments^32^.

Variant calling by GATK 4.2, showed various types of SNVs (Fig. 4d) with a very high frequency of insertions. A remarkable point of *Salinibacter ruber* variation was the intra-species insertions originating from the *Haloquadratum walsbyi* and the high frequency of intra-species plasmid integration. Our results are in agreement with those of González-Torres and Gabaldón^32^, who reported a highly variable accessory genome in *Salinibacter ruber* and highlighted the impacts of horizontal gene transfer (HGT) and homologous recombination (HR) processes^32^.

LUM.80 is taxonomically affiliated to *Salinibacter ruber* ST67 with ANI (Average nucleotide identity) of 98.60% (and to *Salinibacter ruber* SP2521 with ANI of 98.52%), with a G+C content of 67.26%, completeness of 75.87%, and relative abundance of 1.25%, which its overall variation is lower than that of *Salinibacter ruber* ST67 (Fig. 4e).

Other genomes detected in the metagenome have a relative abundance of lower than 1% and were not the focus of our analysis to explore their genomic variations.

### DNA exchange mechanisms in Lake Urmia prokaryotes

Three major mechanisms of DNA exchange in prokaryotes include natural transformation, transduction, and conjugation^33^. To assess the role of transduction in DNA exchange in Lake Urmia we used VirSorter2^34^. VirSorter2^34^ provided viral sequences that made up a small proportion (0.063%) of total sequences within the Lake Urmia metagenome (Supplementary Table S9). In total, 40 viral sequences (including 24 double-stranded DNA phage and 16 single-stranded DNA viruse affiliated ccontigs) were identified, among which 21 sequences could be taxonomically classified by using the genome detective virus tool (v 1.133). A total of 15 viral sequences were similar to previously reported haloviruses and accounted for 0.013% of the total metagenome. In addition, two *Natrialba* phage PhiCh1, two Archaeal BJ1 virus, one *Haloarcula hispanica* pleomorphic phage, and one *Halorubrum* phage HF2, accounting for 0.002% of the metagenome were identified. Nineteen viral sequences (accounting for 0.048% of the metagenome) were not assigned to other previously reported viruses. To evaluate the host-virus relationships, tRNAscan-SE 2.0, PHASTER (Enhanced Release), and minCED (0.4.2) tools detected no tRNA and CRISPR sequences within the LUMs presenting no evidence for host prediction and limited our ability to further explore the role of transduction.

To assess the prevalence of conjugation, insertion sequences were evaluated for their affiliation to plasmid sequences. Although some plasmid sequences were identified, the very short size of these sequences suggests a limited possibility of whole plasmid conjugation.

For transformation, as described earlier, stable DNA fragments present in Lake Urmia could potentially pass through the cell envelope, composing of the S layer and the cell wall. A high concentration of cations can react with the negative charges of the cell envelope, increasing its stability and reducing permeability to prevent DNA entry^35^. However, during chromosome duplication and cytoplasm separation, the hydrolytic enzymes such as pseudomurein endoisopeptidases PeiW and PeiP determined in *Methanothermobacter* strains and archaeosortase detected in all archaea (KO K24447, also detected in Lake Urmia metagenome) degrade the cell envelope, causing cells to have an absent or defected cell envelop. This could be posed as an opportunity for DNA to bypass the cell envelope and transfect the cell^36^. When entering the cell, DNA can be a substrate for restriction enzymes. However, specific restriction enzymes of the cells can distinguish exogenous DNA by considering specific DNA methylation patterns of the host. Host-mimicking DNA (possessing the same DNA methylation pattern) can cause the exogenous DNA not to be recognized by the restriction enzymes. The nucleotide similarity of the exogenous DNA can be a signal to recruit the ubiquitous RecA instead of the nucleases. The incoming exogenous DNA can be replaced in the host genome by homologous recombination^37^. Because DNA present in the lake originates from strains of a few genera with similar methylation pattern, in case of insertion into the living cells, they may be used as beneficial sources to repair the damaged DNA sequences of the host cell via recombination^37^. This hypothesis is consistent with our results. As shown in Supplementary Table S8, a highly significant number of the insertions in *Haloquadratum walsbyi, Halovenus*, and *Salinibacter ruber* originated from various strains of the species (most likely with similar methylation patterns)^38^. The other immunity mechanism is the CRISPR–Cas in which the base pair complementarity of the crRNA with the exogenous DNA triggers nucleases to degrade foreign DNA^38^. However, if there is even a point mutation (which is abundant in the genomes of the Lake Urmia) or base substitution (like cytosine to 5-hydroxymethylcytosine) the complementarity structure is not formed resulting in nullifying the CRISPR–Cas^39^. We presented some bypassing mechanisms of the host cell immune response but regarding the length of the insertions (up to 354 nucleotides), it seems that the exogenous DNA has been unsuccessful in evading the immunity system of the cell. To explain this, it can be hypothesized that the exogenous DNA is either degraded in the environment to smaller sequences that can pass the membrane to be integrated, or the large DNA sequences can enter the cell but are degraded to small sequences before integration. Given that small DNA sequences can pass the membrane more efficiently, the former hypothesis may better illustrate the transformation mechanism in Lake Urmia.

### Functional assessment of the core and accessory genes of *Haloquadratum walsbyi*

The sequences representing the core and accessory clusters of *Haloquadratum walsbyi* were 576 and 178 KOs (organized in Orthologs, modules, and networks), respectively. Annotated genes of the core components could be organized in 11 KEGG modules covering all essential proteins in carbohydrate, energy, nucleotide, amino acid, cofactors, vitamins, terpenoids, and polyketides metabolism (Supplementary Table S12). The genes in the auxiliary components did not form any complete module. Our result showed that the genes involved in the prokaryotic defense system (restriction and modification system and toxin–antitoxin system) and RNA polymerase are exclusively present in the core signifying their crucial roles. Restriction and modification systems include type I restriction enzyme, DNA methyltransferases, adenine-specific DNA-methyltransferase, and modification methylase. Detailed KEGG analysis of the core and auxiliary components is presented in Supplementary Tables S10–S12. Further, various chaperonins including heat shock proteins HSP60, heat shock proteins GimC, folding catalyst dnaJ (which play a role in stress response), and the proteasome responsible for folding, sorting, and degradation of the mistranslated, misfolded, and damaged proteins^40^ are mostly organized in the core.

## Conclusion

To understand the community structure and microdiversity of Lake Urmia (as an extreme habitat with a high concentration of ions), we reconstructed MAGs and analyzed various modes of genetic variation in them. In terms of horizontal gene transfer processes, transformation was detected to be the main strategy of DNA insertion. Our analysis showed that bacteria and archaea exhibit high inter/intra-genera gene exchange and metagenomic analysis revealed a high abundance of *Haloquadratum walsbyi*, *Halonotius*, and *Salinibacter ruber* representing high SNVs leading to challenges in reconstructing complete MAGs from their representatives in the Lake Urmia metagenomes due to the very high level of microdiversity. Finally, we suggest that the high ionic concentrations of such hypersaline ecosystems might play a role in the microdiversity profile of its highly abundant taxa where more empirical analyses will be required to fully clarify its role.

## Methods

### Geographic description of the study site, physico-chemical analysis, and sampling procedures

The lake is located between the provinces of East Azerbaijan and West Azerbaijan in Iran, and West of the southern portion of the Caspian Sea. In coordination with the Iranian Department of Environment, samples were collected on October 20th, 2020. Approximately 20 L of water was collected in sterile containers at 20 cm depth from six different locations along the vertical transect of Lake Urmia as is shown in Fig. 1. Due to the very low water levels in the southern part of the lake, no sample was collected from this part. Samples were kept cold until further analysis. For physico-chemical analysis of the samples, standard methods based on American Public Health Association (APHA) were used^41^.

### Environmental DNA extraction and quality control

To collect biomass for metagenomic sequencing, we removed larger particles and eukaryotes such as *Dunaliella salina,* by pre-filtering samples through 3-μm filters (cellulose-nitrate, Millipore). For environmental DNA extraction, we mixed the water samples (120 L) and used 40 L for biomass collection using two strategies. Ten liters of the sample were centrifuged at 4500 rpm (3260×*g*) for 60 min, and retentate was used for DNA extraction using QIAprep® Miniprep (Qiagen). The second strategy was biomass collection on polycarbonate membrane filters with 0.22-μm pore size (Isopore Membrane Filter, Isopore™ Millipore), and finally DNA extraction from filters using Qiagen DNeasy (Qiagen). The quantity and quality of the extracted DNA were analyzed by a NanoDrop™ One C Microvolume UV–Vis Spectrophotometer and agarose gel electrophoresis.

### Sequencing and assembly

The purified environmental DNA was sequenced using Illumina NovaSeq 6000 platform at Novogene Co. Ltd (China) as a paired-end (PE150) library. Metagenomic raw reads were quality-checked using FastQC 0.11.9^42^ and trimmed using Trimmomatic^43^. The paired-end sequences were merged using BBtools, reformat.sh script (sourceforge.net/projects/bbmap/). For preliminary taxonomic profiling, the raw reads of the metagenomic sample were analyzed using MetaPhlAn3^15^ with a database of 1.1 M markers using bowtie2^44^. The trimmed read sets were assembled using MEGAHIT (v1.0.3)^45^ with paired-end mode, k min = 49, k max = 149, k step = 10. To evaluate assembly quality, QUAST^46^ was applied. For metagenome diversity, rarefaction analysis based on phylogenetic reads was performed using MG-RAST server 4.0.3^47^.

### Detection and phylogenetic analysis of ribosomal RNA in the metagenome assemblies

To retrieve ribosomal RNA genes from the metagenomic assemblies, barrnap v0.9^16^ was used which predicts the location and sequence of ribosomal RNA genes in genomes and supports both Bacteria (5S, 23S, and 16S rRNA) and Archaea (5S, 5.8S, 23S, and 16S rRNA). The 16S rRNA sequences were checked against three databases including NCBI, EzBioCloud, and the genome taxonomy database (GTDB) 16S rRNA sequences (release 202) using NCBI-BLAST+PACKAGE. For phylogram construction, Ngphylogeny.fr^48^ followed by iTOL v6^49^ were used.

### Reconstructing Lake Urmia MAGs, quality check, and taxonomy assignment

Binning of assembled contigs for reconstructing metagenome-assembled genomes (MAGs) was carried out for contigs ≥ 2.5 kb using MetaBAT2^50^. Mapping metagenomic reads to the assembly were performed using Bowtie2^44^, with the setting (-local-sensitive). The completeness and contamination of the reconstructed Lake Urmia MAGs (abbreviated as LUM) were evaluated using CheckM (v1.1.3)^51^. LUMs with CheckM-completeness ≥ 40% and contamination < 5% were selected for further analysis. For contamination estimation by 16S rRNA genes and other statistics, ContEst16S^52^ was used. Taxonomic assignment of the LUMs was performed using GTDB-tk v1.3 (Pierre-Alain Chaumeil, 2020) and average nucleotide identities were calculated using FastANI v1.33^53^. Additionally, 16S rRNA gene sequences were extracted from representative LUMs using Barrnap v0.9 and analyzed using NCBI-BLAST+PACKAGE. To map the community structure of the metagenome, Bowtie 2, using -local-sensitive setting was used to a reference genome file containing all 22 LUMs, and publicly available genomes including 132 bacterial, 152 archaeal, and 10 viral genomes (in total 294 genomes). Results were processed using SAMtools-1.14 followed by CoverM (0.6.1) to analyze the community structure of the Lake Urmia metagenome.

### Identification of viral contigs

To retrieve viral contigs associated with the metagenome and the LUMs, we used VirSorter2^34^. Viral sequences with a max score of 1.00 underwent the following analyses. For the taxonomic assignment, the retrieved sequences were analyzed by genome detective virus tool version 1.133 (which is based on the viral Refseq protein database from NCBI). tRNAs were analyzed with tRNAscan-SE 2.0^54^, for CRISPR evaluation, viral sequences were assessed by the minCED (0.4.2)^55^, and PHASTER (PHAge Search Tool-Enhanced Release) for phage identification^56^.

### Microdiversity profiling

Microdiversity of reconstructed LUMs and reference genomes were analyzed from the metagenome using two packages. The Genome Analysis Toolkit’s 4.2 HaplotypeCaller^57^ was used for variant discovery and the GVCFs output containing SNVs and INDELs were genotyped by GATK GenotypeGVCFs. Insertion sequences were identified using NCBI-BLAST+PACKAGE. For population-level diversity, inStrain 1.5.7 was used, by which, the metagenome is mapped to the reference genome (mentioned in the previous section) to calculate each gene, scaffold, and/or genome average nucleotide identity (ANI), depth and breadth of coverage, SNP (SNV, and SNS), and major allele frequencies of bi-allelic SNVs^5^.

### Pan-genome evaluation

Pan-genome analysis of the *Haloquadratum walsbyi* C23, *Haloquadratum walsbyi* DSM 16790, and LUM.22, was carried out using SuperPang^58^ with an identity threshold of 0.95. For functional evaluation, the KEGG Orthology (ko) identifiers of the PROKKA annotated genes of the core and auxiliary clusters were retrieved by KEGG BlastKOALA^59^, and the modules were reconstructed using KEGG Mapper-Reconstruct^59^.

## Supplementary Information

Supplementary Figure 1.

Supplementary Figure 2.

Supplementary Table 1.

Supplementary Table 2.

Supplementary Table 3.

Supplementary Table 4.

Supplementary Table 5.

Supplementary Table 6.

Supplementary Table 7.

Supplementary Table 8.

Supplementary Table 9.

Supplementary Table 10.

Supplementary Table 11.

Supplementary Table 12.

## Data Availability

The Lake Urmia metagenome and metagenome-assembled genomes (MAGs) reconstructed in this study can be accessed under the BioProject accession PRJNA825141 and the following accession link: https://www.ncbi.nlm.nih.gov/bioproject/PRJNA825141.
