# Publisher Correction: Hypersaline Lake Urmia: a potential hotspot for microbial genomic variation

**DOI:** 10.1038/s41598-023-29691-w

**Published:** 2023-02-10

**Authors:** Roohollah Kheiri, Maliheh Mehrshad, Ahmad Ali Pourbabaee, Antonio Ventosa, Mohammad Ali Amoozegar

**Affiliations:** 1 Extremophiles Laboratory, Department of Microbiology, School of Biology and Center of Excellence in Phylogeny of Living Organisms, College of Science, University of Tehran, Tehran, Iran; 2 Department of Aquatic Sciences and Assessment, Swedish University of Agricultural Sciences, 750 07 Uppsala, Sweden; 3 Department of Soil Science, Agriculture Engineering and Technology, College of Agriculture and Natural Resources, University of Tehran, Karaj, Iran; 4 Department of Microbiology and Parasitology, Faculty of Pharmacy, University of Sevilla, 41012 Sevilla, Spain

Correction to: *Scientific Reports* 10.1038/s41598-023-27429-2, published online 07 January 2023

The original version of this Article contained errors.

In the Introduction,

“Studies on different hypersaline environments show that Haloquadratum and certain Balneolaeota members may preferably grow in aquatic or soil habitats, respectively, while hanohaloarchaea, nanohaloarchaea, and Salinibacter are capable of adapting to both environments^3^.”

now reads:

“Studies on different hypersaline environments show that Haloquadratum and certain Balneolaeota members may preferably grow in aquatic or soil habitats, respectively, while haloarchaea, nanohaloarchaea, and Salinibacter are capable of adapting to both environments^3^.”

In addition, in the Results and discussion section, under the subheading ‘Genus *Salinibacter*’,

“Our results are in agreement with those of Gonzálezó and Gabaldón^32^, who reported a highly variable accessory genome in *Salinibacter ruber* and highlighted the impacts of horizontal gene transfer (HGT) and homologous recombination (HR) processes^32^.”

now reads:

“Our results are in agreement with those of González-Torres and Gabaldón^32^, who reported a highly variable accessory genome in *Salinibacter ruber* and highlighted the impacts of horizontal gene transfer (HGT) and homologous recombination (HR) processes^32^.”

The original Article has been corrected.

