# Supplementary figures and images for: Hypersaline Lake Urmia: a potential hotspot for microbial genomic variation
Source: Sci Rep. 2023 Jan 7;13:374. doi: 10.1038/s41598-023-27429-2 (PMC9825399; doi:10.1038/s41598-023-27429-2)

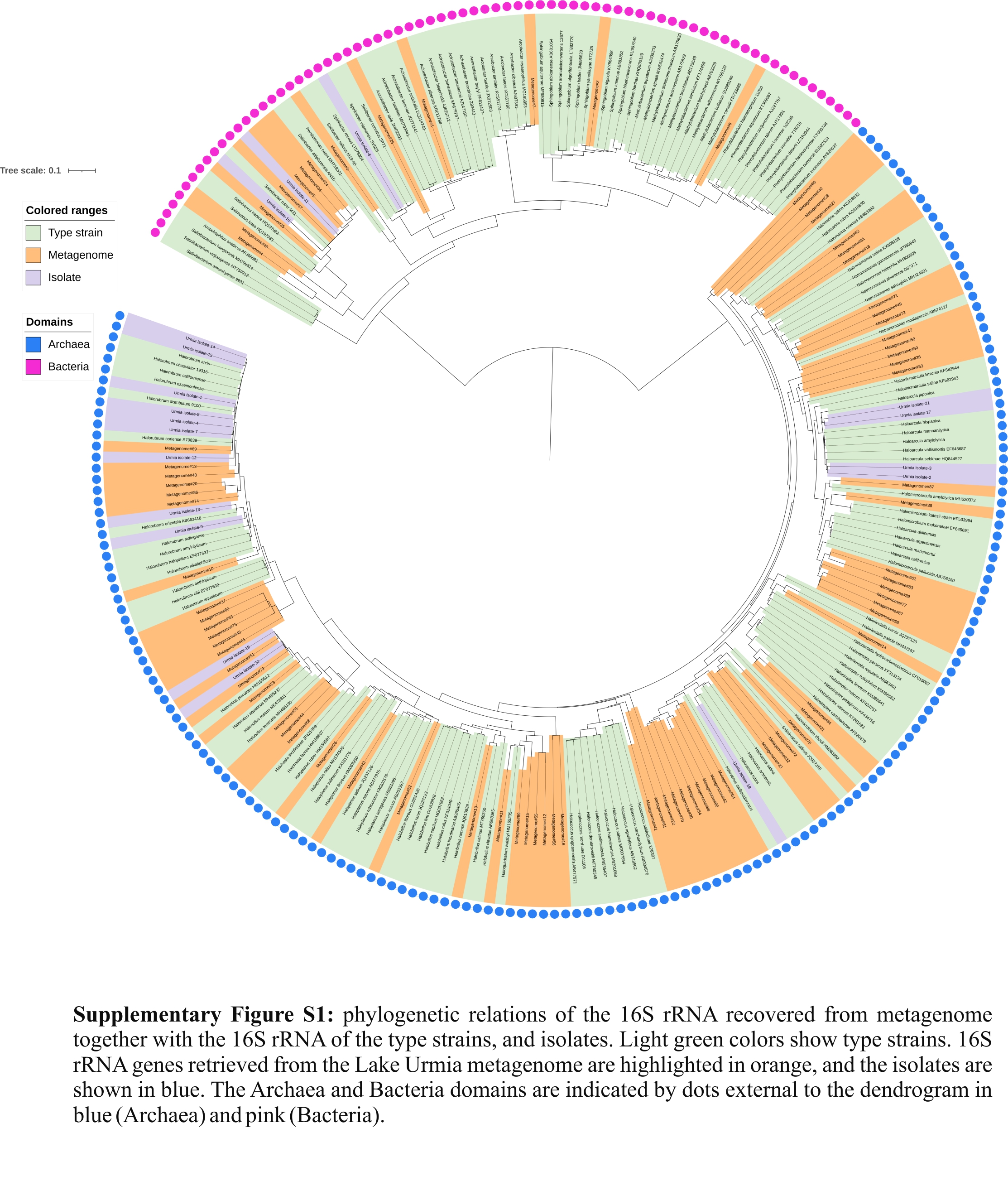

Supplement: Supplementary file 1 — Supplementary Figure 1. [file 41598_2023_27429_MOESM1_ESM.jpg]
