# Supplementary material for: Hypersaline Lake Urmia: a potential hotspot for microbial genomic variation
Source: Sci Rep. 2023 Jan 7;13:374. doi: 10.1038/s41598-023-27429-2 (PMC9825399; doi:10.1038/s41598-023-27429-2)
Supplement: Supplementary file 2 — Supplementary Figure 2. [file 41598_2023_27429_MOESM2_ESM.docx]

***Halonotius* sp. J07HN6 (3.96%)**

***Salinibacter ruber* strain ST67 (1.19 %)**

***Uncultured archaeon* A07HR60 (1.64 %)**

**LUM.22 *(Haloquadratum walsbyi)* (11.69%)**

***Haloquadratum walsbyi* DSM 16790 (14.53%)**

***Halonotius* sp. J07HN4 *(*1.20%)**

**LUM.78 (*Halovenus*) *(*1.00 %)**

**LUM.80 (*Salinibacter ruber*) *(*1.26 %)**

**Unmapped (28.23%)**

**LUM.45 *(Halovenus) (*1.94%)**

***Halonotius pteroides strain* CECT 7525 (6.8%)**

**LUM.44 (*Salinirussus*) *(*3.2%)**

**LUM.12 (*Haloferacaceae*) *(*9.39%)**

**Supplementary Figure S2:** Relative abundance of reference genomes and LUMs in the Lake Urmia metagenome.
